# Genetics in inclusion body myositis

**DOI:** 10.1097/BOR.0000000000000431

**Published:** 2017-09-15

**Authors:** Simon Rothwell, James B. Lilleker, Janine A. Lamb

**Affiliations:** a Centre for Musculoskeletal Research, Division of Musculoskeletal and Dermatological Sciences, Faculty of Biology, Medicine and Health, Manchester Academic Health Science Centre, The University of Manchester, Manchester; b Greater Manchester Neurosciences Centre, Manchester Academic Health Science Centre, Salford Royal NHS Foundation Trust, Stott Lane, Salford; c Centre for Epidemiology, Division of Population Health, Health Services Research and Primary Care, Faculty of Biology, Medicine and Health, Manchester Academic Health Science Centre, The University of Manchester, Manchester, UK

**Keywords:** degeneration, genetic association study, genetics, inclusion body myositis, mitochondria, sequencing

## Abstract

**Purpose of review:**

To review the advances in our understanding of the genetics of inclusion body myositis (IBM) in the past year.

**Recent findings:**

One large genetic association study focusing on immune-related genes in IBM has refined the association within the human leukocyte antigen (HLA) region to *HLA-DRB1* alleles, and identified certain amino acid positions in HLA-DRB1 that may explain this risk. A suggestive association with *CCR5* may indicate genetic overlap with other autoimmune diseases. Sequencing studies of candidate genes involved in related neuromuscular or neurodegenerative diseases have identified rare variants in *VCP* and *SQSTM1.* Proteomic studies of rimmed vacuoles in IBM and subsequent genetic analyses of candidate genes identified rare missense variants in *FYCO1*. Complex, large-scale mitochondrial deletions in cytochrome c oxidase-deficient muscle fibres expand our understanding of mitochondrial abnormalities in IBM.

**Summary:**

The pathogenesis of IBM is likely multifactorial, including inflammatory and degenerative changes, and mitochondrial abnormalities. There has been considerable progress in our understanding of the genetic architecture of IBM, using complementary genetic approaches to investigate these different pathways.

## INTRODUCTION

Sporadic inclusion body myositis (IBM) is the most common acquired muscle disease presenting in people over 50 years of age. Clinically, it is characterized by slowly progressive weakness and muscle wasting predominantly of the quadriceps and long finger flexor muscles.

In IBM, inflammatory features in muscle biopsy specimens suggest an immune-mediated component to disease pathogenesis. In addition, circulating anti-Ro autoantibodies may be found in around 20% of patients and recent work has identified cytosolic 5′-nucleotidase 1A (anticN-1A) autoantibodies in around one-third of patients. However, unlike other idiopathic inflammatory myopathies (IIMs), such as polymyositis and dermatomyositis, IBM is unresponsive to conventional immunosuppressive treatments. This lack of response may be explained by evidence of impaired autophagic processes, including rimmed vacuole formation and the accumulation of misfolded proteins. Whether these degenerative processes represent a primary or secondary involvement is unclear.

Some hereditary diseases may mimic clinical features of IBM. These diseases may also exhibit similar pathological features, such as rimmed vacuoles and protein accumulations [1,2]. These disorders are sometimes referred to as hereditary IBM (hIBM), but are better described using the associated genetic abnormality. Genes involved with these ‘rimmed vacuolar myopathies’ will not be discussed here, but have been reviewed previously [3,4].

Although the primary cause of IBM remains unknown, genetic factors likely influence disease susceptibility. This article reviews advances that have been made in the past year in our understanding of the genetic component of IBM and potential future approaches for research in this rare disease. 

**Box 1:**
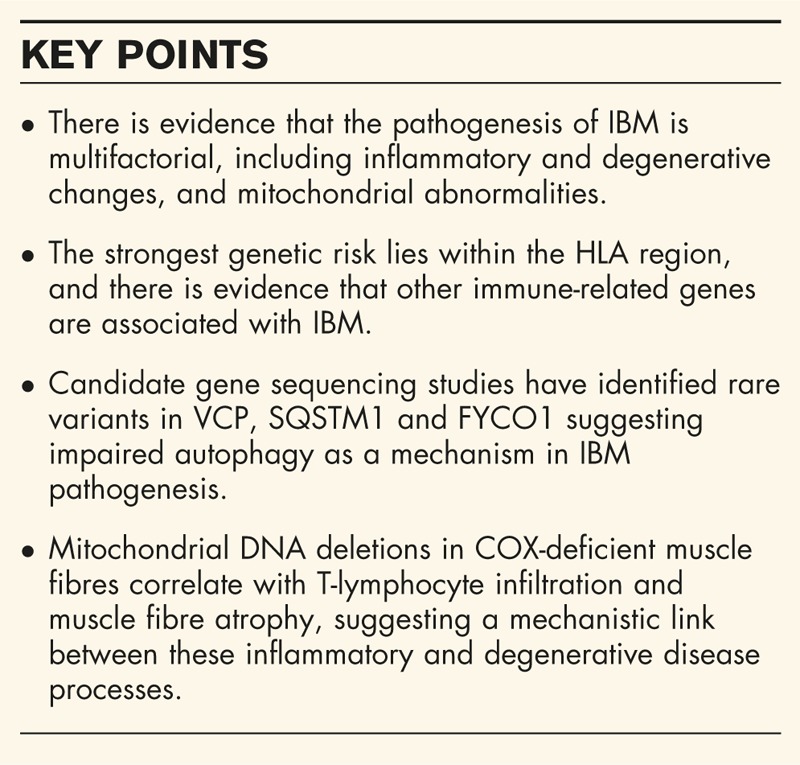
no caption available

## APPROACHES IN GENETIC STUDIES

There has been considerable progress in our understanding of the genetic basis of IBM. Two different approaches have been used recently in IBM; large genetic association studies and smaller targeted sequencing studies.

**FIGURE 1:**
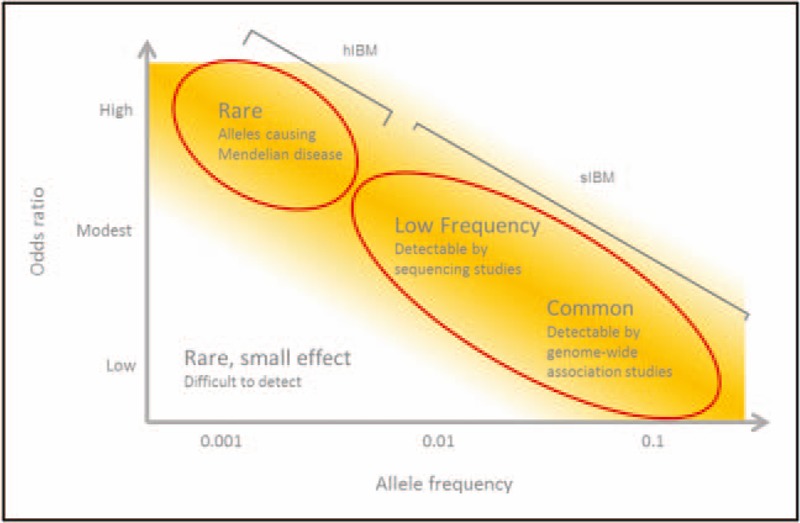
Strategies for identifying genetic variants in IBM. Rare causal variants of high effect size are expected in Mendelian diseases such as hIBM. Genetic variants contributing to IBM susceptibility are expected to have a more modest effect size. Low-frequency variants of intermediate effect sizes will likely be found using sequencing studies. Common variants of low effect size will be detectable by well-powered genetic association studies. hIBM, hereditary IBM; IBM, body myositis.

The study design of genome-wide association studies (GWAS) and contemporary genetic association studies frequently test hundreds of thousands, if not millions, of single nucleotide polymorphisms (SNPs) across the genome. Results from GWAS in autoimmune diseases suggest that most associated variants reside in regulatory regions, exerting their low effect sizes (odds ratio <1.1) on the expression of immune-mediated genes [5,6]. The combination of the burden of multiple testing and modest effect sizes of associated SNPs means that studies in rare diseases are hampered by low power because of small sample size. Success will depend on the presence of common variants of modest effect sizes (Fig. 1).

Other approaches to investigate the genetic component of IBM have relied on detecting rarer variants through sequencing studies that commonly involve fewer individuals than GWAS. To date, these have focused on candidate genes taken from related neuromuscular or neurodegenerative diseases. However, novel approaches are also being used, including targeting of genes identified in proteomic studies. Variants discovered through these studies likely will be rarer, with a larger effect size than those discovered by GWAS. Therefore, GWAS and sequencing approaches test complementary hypotheses to investigate the genetic architecture of IBM.

## IMMUNE-RELATED GENES IN INCLUSION BODY MYOSITIS

There are multiple lines of evidence for inflammation as a key pathway in the pathogenesis of IBM. These include the presence of CD8^+^ cytotoxic T cells surrounding major histocompatibility complex (MHC) class I-expressing fibres [7], the presence of plasma cells within affected muscle [8], IBM specific and nonspecific autoantibodies [9,10] and a strong genetic association with the human leukocyte antigen (HLA) region [11–13]. Recent studies suggest that several autoimmune diseases, including the IIMs, share genetic overlap for susceptibility to disease [14,15]. In line with this evidence, a recent study hypothesized that there may be shared immune loci associated with IBM [16^▪▪^].

Through the international Myositis Genetics Consortium (MYOGEN), 252 patients with IBM were recruited and genotyped on the Illumina Immunochip array. This SNP array contains coverage of 186 established autoimmune susceptibility loci and extended coverage across the MHC. The strongest associations with IBM were seen within the MHC, therefore imputation was used specifically to investigate classical class I and class II HLA alleles that may be explaining the risk in this region. Three HLA-DRB1 alleles were found to be independently associated with IBM; HLA-DRB1^∗^03 : 01, HLA-DRB1^∗^01 : 01 and HLA-DRB1^∗^13 : 01. Although HLA-DRB1^∗^03 : 01 is known to be associated with polymyositis and dermatomyositis, the association with HLA-DRB1^∗^01 : 01 and HLA-DRB1^∗^13 : 01 is unique to IBM within the IIMs. Unlike many other diseases [14,17,18], it is interesting to note that the association of IBM with HLA is localized to HLA-DRB1. Although this may be due to low power to detect other HLA genes, it is in keeping with previous studies in IBM [12,19]. One potential explanation for risk shared across multiple HLA alleles is an amino acid ‘signature’ that may confer risk. When analyzing amino acid positions within HLA-DRB1, positions 26 and 11, located within the peptide binding groove, were associated with IBM. The strongest associations were with amino acids present on classical risk haplotypes, such as a tyrosine at position 26, predominantly carried on HLA-DR3 alleles. Functional research is needed to elucidate whether the association within this gene can be explained by these amino acids. Other candidate genes within the MHC were not investigated in this study, for example *NF-kB* genes, *TNFA* and *NOTCH4*[20–22]. *NOTCH4* has been associated previously with IBM, although because of the strong linkage in this region, it is not clear whether it is directly involved in disease or associated because of carriage on classical HLA-risk haplotypes.

Potential genetic associations within the HLA region were also investigated for the development of anticN-1A (NT5c1A) antibodies, a recently described autoantibody common in IBM. A significant association was seen with HLA-DRB1^∗^03 : 01 when compared to healthy controls; however, there was no difference when 35 anticN-1A positive patients were compared to 68 anticN-1A negative IBM patients [16^▪▪^]. This suggests that there is no strong HLA association with the antibody over and above the general association with HLA-DRB1^∗^03 : 01 in IBM. Similarly, a study in 24 anticN-1A positive IBM patients did not detect a unique HLA class II association independent of HLA-DR3 [23].

In the total IBM analysis, no loci outside the MHC reached genome wide significance [16^▪▪^]. However, three other loci reached a suggestive level of significance, one of which was assigned to the *CCR5* gene. The authors hypothesize that the protective effect of this association may be due to a frameshift mutation in *CCR5* which inhibits its function as a chemokine receptor involved in T-cell migration. *CCR5* previously has been associated with other autoimmune diseases lending support to the hypothesis of an immune-mediated component to IBM [24].

## SEQUENCING STUDIES IN INCLUSION BODY MYOSITIS

Evidence for a degenerative component of IBM pathogenesis includes the formation of rimmed vacuoles and accumulation of misfolded proteins such as β-amyloid, p62, TDP43 and phosphorylated tau. To date, candidate gene studies mostly have focused on genes known to be associated with neurodegenerative diseases such as Alzheimer's disease, Parkinson's disease and amyotrophic lateral sclerosis (ALS). Genes investigated include amyloid β precursor protein, microtubule-associated protein tau, α-1-antichymotrypsin (*SERPINA3*), prion protein and *C9orf72.* However, these studies have failed to find significant associations [4]. A well-studied locus is Apolipoprotein E (*APOE*) -‘translocase of outer mitochondrial membrane 40’ (*TOMM40*) [25–27], and although a recent study showed no significant associations with risk of developing IBM, a potential association with later onset of symptoms was reported [28]. Another negative study investigated genetic variants within autoantibody targets such as *cN1A* or *cN1B*[29]. One large candidate gene sequencing study reported, among others, two rare variants in the valosin-containing protein gene (*VCP*) [30]. Variants in *VCP* cause IBM associated with Paget's disease of the bone (PDB) and frontotemporal dementia (FTD); however, neither patient in this study manifested other symptoms reported with VCP mutations and both fulfilled the diagnostic criteria for IBM.

A recent study used whole exome sequencing (WES) in 181 IBM patients focusing on p62, also named sequestosome 1 (SQSTM1), and *VCP* genes, both of which are known to harbour genetic variants associated with ALS, PDB and FTD [31^▪▪^]. They report four rare missense variants in *SQSTM1* and three variants in *VCP.* This represented 4.0% of the cohort, and is the first time potential pathogenic variants in *SQSTM1* have been observed in IBM patients. As these variants may cause diseases that mimic IBM, it was confirmed that none of these patients had developed symptoms of PDB, FTD or ALS and all fulfilled diagnostic criteria for IBM. As *SQSTM1* is involved in the autophagy pathway, and *VCP* is involved in proteasomal degradation of misfolded proteins, this further supports the role of autophagic alterations and aggregation of proteins in the pathogenesis of IBM. These studies suggest that there is merit in the targeted sequencing of genes previously associated with hIBM and other inherited muscle disorders.

Rather than using related diseases, a novel way of identifying candidate genes is using proteomic analysis. We know that p62/SQSTM1 accumulates in inclusions of IBM muscle fibres [32]. A recent study sought to identify other proteins present in the rimmed vacuoles in skeletal muscle of IBM patients by mass spectrometry [33^▪▪^]. Two hundred and thirteen proteins showed a statistically significant overrepresentation in rimmed vacuole samples compared to controls. Many proteins already known to be involved in IBM or other protein aggregate myopathies overlap with the proteins identified, validating this approach. The 173 novel proteins not described before in IBM warrant further investigation. Proteins that were present in at least 50% of rimmed vacuole samples (131 genes) were taken forward for genetic analysis using WES data from 62 patients with IBM. Hundred missense or loss of function variants were identified in 52 genes. Genetic data from ALS patients were then used to identify variants statistically enriched that are specific to IBM. Rare missense or loss of function variants in *FYCO1* were enriched in IBM patients (11.3%) compared to ALS patients (2.6%, *P* = 0.003) or healthy controls (3.4%, *P* = 0.01). *FYCO1* is involved in autophagosome/endosome trafficking. Along with the *VCP* and *SQSTM1* associations described above, this provides further evidence for autophagosome processing as a basis for future mechanistic studies. Novel treatments targeting protein dyshomeostasis are currently in development [34].

In contrast to the studies outlined above, a smaller sequencing study from Finland did not find any rare missense genetic variants [35]. This study sequenced the exomes of 30 patients from Finland and a replication cohort of 12 patients from Italy, with the hypothesis that the genetically more homogeneous Finnish population would be conducive to identifying genetic risk variants. Initially, a candidate gene approach on WES data was taken, focusing on 180 genes including those known to cause hereditary primary myopathies as well as 42 novel candidate genes [36]. No rare missense variants in *SQSTM1* and *VCP* were found, or in genes that would explain the observed clinical phenotype. A subsequent case−control association analysis identified seven SNPs enriched in the Finnish IBM population with *P* < 0.005. Reassuringly, two of these were within the HLA region; however, the other associations were in novel genes; *STARD3*, *SGPL1* and *SETD4*. Results from a small association study are to be treated with caution, and will need to be replicated.

As discussed above, associations with *VCP*, *SQSTM1* and *FYCO1* validate the use of a sequencing approach, and have given us greater mechanistic insight in to IBM cause. It is worth noting, however, that in all of these cases, patients lacked a family history of IBM or weakness, indicating that these inherited variants alone are not sufficient for disease pathogenesis. There are likely many more variants that predispose to IBM. Identification of novel genes will come from WES and whole genome sequencing (WGS) studies that detect rarer coding variants with potentially larger effect size. Additional WES studies in IBM are currently being undertaken [37]. Future studies will employ WGS, which comprehensively covers the genome including regulatory regions, intronic regions and structural variations that may be missed by WES. WGS studies, however, are still expensive, with the huge amount of data produced being complex, and computationally expensive to analyse and store.

## MITOCHONDRIAL DNA DELETIONS

Cytochrome c oxidase (COX)-deficient muscle fibres are a common histopathological feature of IBM, and there is increasing evidence that mitochondrial abnormalities may play a role in the cause of this disease. Recent research has shown increased mitochondrial DNA (mtDNA) deletions in COX-deficient muscle fibres, and that the proportion of these deficient fibres correlates with severity of T-lymphocyte infiltration and muscle fibre atrophy suggesting a mechanistic link between these disease processes [38,34]. In addition, a recent study in IBM has implicated a number of nuclear DNA genes that are associated with transcription, replication and maintenance of mtDNA [39].

Myofibres may harbour clonally expanded large-scale mtDNA deletions responsible for respiratory chain deficiency that is thought to be central to IBM pathogenesis [40,41]. Previous works reporting mtDNA deletions have been based on a small number of mtDNA genes, and were focused on single major arc deletions. A recent study sought to characterize mtDNA deletions in more detail in patients with IBM [42^▪^]. mtDNA rearrangements were investigated in single muscle fibres from patients with IBM using complementary techniques. The authors confirmed the presence of mtDNA deletions in 78 out of 92 (∼85%) COX-deficient cells, but demonstrated that mtDNA rearrangements in IBM are more complex than previously assumed. The authors showed for the first time that 20% of COX-deficient cells harboured two or more mtDNA deletions. In addition, some unusually large deletions were detected that extended into the origin of light strand replication.

Further evidence for mitochondrial dysfunction in IBM is provided by research that sought to characterize mitochondrial phenotype at a genetic, molecular and functional level in 30 IBM patients [43^▪^]. The authors also compared mitochondrial differences between muscle and peripheral blood mononuclear cells (PBMCs) in IBM cases and controls to assess whether these alterations could be used as biomarkers in a less invasive tissue. Multiple mtDNA deletions were found in the muscle of 57% patients, and while there was a significant decrease in total mtDNA in muscle, the decrease was less pronounced in PBMCs. This may be attributed to shorter lifespan of PBMCs and thus reduced accumulation of mitochondrial deficiencies. The authors found that mitochondrial COX activity was decreased in both muscle and PBMCs suggesting that mitochondrial dysfunction may not be confined to the target tissue of the disease.

## CONCLUSION

**FIGURE 2:**
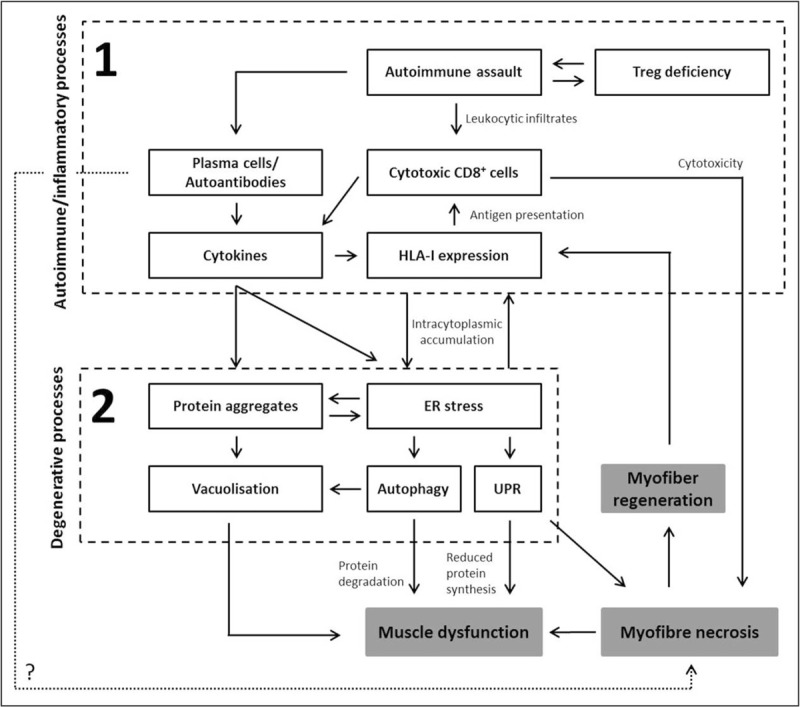
A proposed model for the pathogenesis of IBM. Inflammation within muscle (Box 1) may induce fibre injury and HLA-I overexpression. Overloaded protein degradation systems (Box 2) induce misfolded protein deposits in muscle fibres. ER, endoplasmic reticulum; UPR, unfolded protein response. Figure adapted from [44].

There has been considerable progress in our understanding of the genetic architecture of IBM. Evidence suggests a complex interplay between inflammatory and degenerative processes and mitochondrial abnormalities. A model starting with inflammation within muscle and subsequent deposition of amyloid and other proteins because of overloaded protein degradation systems has been proposed by Benveniste *et al.* in Fig. 2[44]. Complementary approaches to investigate these hypotheses have been used successfully in IBM. Investigating the effect of common variants on disease susceptibility in rare diseases will rely in part on continuing sample collection by coordinated international collaborations, including different ethnicities that will facilitate larger studies. In addition, novel statistical methods are being developed that leverage the power from larger datasets in related diseases because of the similarities in genetic susceptibility [45]. WES and WGS studies will identify novel variants across the genome and may uncover previously overlooked biological processes to further expand our knowledge of the genetic component of IBM.

## Acknowledgements

None.

### Financial support and sponsorship

This work was funded by an MRC Partnership Grant (MR/N003322/1).

### Conflicts of interest

There are no conflicts of interest.

## REFERENCES AND RECOMMENDED READING

Papers of particular interest, published within the annual period of review, have been highlighted as:

▪ of special interest
▪▪ of outstanding interest
